# Impact of Impulses on Microstructural Evolution and Mechanical Performance of Al-Mg-Si Alloy Joined by Impulse Friction Stir Welding

**DOI:** 10.3390/ma14020347

**Published:** 2021-01-12

**Authors:** Iuliia Morozova, Aleksei Obrosov, Anton Naumov, Aleksandra Królicka, Iurii Golubev, Dmitry O. Bokov, Nikolay Doynov, Sabine Weiß, Vesselin Michailov

**Affiliations:** 1 Department of Joining and Welding, Brandenburg University of Technology Cottbus-Senftenberg, 03046 Cottbus, Germany; iurii.golubev@b-tu.de (I.G.); nikolay.doynov@b-tu.de (N.D.); michailov@b-tu.de (V.M.); 2 Department of Metallurgy and Materials Technology, Brandenburg University of Technology Cottbus-Senftenberg, 03046 Cottbus, Germany; aleksei.obrosov@b-tu.de (A.O.); weiss@b-tu.de (S.W.); 3 Lightweight Materials and Structures Laboratory, Peter the Great St. Petersburg Polytechnic University, 195251 St. Petersburg, Russia; anton.naumov@spbstu.ru; 4 Faculty of Mechanical Engineering, Wroclaw University of Science and Technology, Wybrzeże Wyspiańskiego 27, 50-370 Wrocław, Poland; aleksandra.krolicka@pwr.edu.pl; 5 Pharmaceutical Natural Sciences Department Institute of Pharmacy, Sechenov First Moscow State Medical University, 8 Trubetskaya St., bldg. 2, 119991 Moscow, Russia; bokov_d_o@staff.sechenov.ru

**Keywords:** Al-Mg-Si alloy, impulse friction stir welding (IFSW), precipitation, microstructure evolution, mechanical properties

## Abstract

Impulse Friction Stir Welding (IFSW) was utilized to join 6082–T6 alloy plates at various impulse frequencies. A distinctive feature of IFSW is the generation of mechanical impulses that enhances the forging action of the tool, and thereby, alters the weld microstructure. The microstructural evolution in the Stir Zone (SZ) with special focus on the strengthening precipitation behavior, and overall mechanical properties of the IFSW joints have been investigated. It was demonstrated that the strengthening β″ precipitates reprecipitated in the SZ of the IFSW joints during natural aging. In contrast, no precipitates were found in the SZ of the Friction Stir Welding (FSW) weld. Partial reversion of β″ after IFSW is supposed to occur due to more developed subgrain network and higher dislocation density introduced by impulses that accelerated precipitation kinetics. Dynamic recrystallisation was facilitated by impulses resulting in a fine, homogeneous structure. There was no significant difference between the microhardness in the SZ, tensile and yield strength of the FSW and IFSW joints. However, the application of impulses demonstrated the smoothing of the hardness reduction in the transition region at the advancing side. The shift of the fracture location from the Heat-Affected Zone (HAZ) by FSW to the SZ as well as higher elongation of the joints by IFSW of lower frequencies could be related to the grain refinement and the change of the grain orientation.

## 1. Introduction

Despite the fact that Friction Stir Welding (FSW) has been widely applied to join the precipitation hardenable aluminum alloys of 6xxx series (Al-Mg-Si), there are several problems associated with precipitation phenomena [1,2,3]. It is known that dispersion hardening in 6xxx alloys is based on the precipitation of the metastable precursors of the equilibrium β-Mg_2_Si particles (could be Mg_5_Si_6_ depending on the chemical composition of the alloy [4,5]). They include Guinier–Preston zone (GP-zone), β″ precipitates, which are characterized by a coherent relationship with a matrix and considered as a main contributor to the material strength, semi-coherent rod-shaped β’ precipitate, and stable β phase. The strengthening β″ precipitates containing in the microstructure of the base 6xxx materials after T6 heat treatment provide the maximum hardening of the alloy. During FSW of Al-Mg-Si alloys such as 6082-T6, these metastable precipitates undergo dissolution and coarsening processes depending on the peak temperature reached in the Stir Zone (SZ), Thermo-Mechanically Affected Zone (TMAZ), and Heat-Affected Zone (HAZ) of the FSWed joints [6,7,8,9,10]. The difference in the local peak temperatures in the transverse direction results in the different volume fraction of the β″ and β’ precipitates formed in the distinct regions.

Previous investigations have revealed that dissolution and transformation of the β″ to β’ precipitates occurred in the HAZ and TMAZ regions (also called transition zone). The formation of β’ phase leads to significant softening and thus degrades the FSW joint efficiency, since β’ possesses only semi-coherency with a matrix and, therefore, is not able to provide as significant hardening as β″ precipitates [1,2,3,11,12]. Based on various studies, it has currently been established that the strengthening β″ precipitates dissolved in the SZ due to the high peak temperature. However, the statements about their reprecipitation during natural aging presented in the literature are controversial. The vast majority of studies, e.g., [3,8,13,14] have not revealed the presence of hardening precipitates in the SZ after welding. There is an assumption that the precipitation response could be suppressed by the high cooling rate, typical of FSW thermal history or by incomplete dissolution. However, the possibility of GP-zone reprecipitation during natural aging is considered to explain the partial increase in hardness in the SZ, together with grain refinement [7,10,12,15]. On the other hand, there is a number of studies that have suggested that strengthening precipitates partially reprecipitated after natural aging or incomplete dissolved in the SZ, leading to the joint strength improvement accompanied by other strengthening mechanisms [4,16,17].

In order to govern the structural transformations in precipitation hardenable aluminum alloys during FSW, e.g., suppress coarsening of the strengthening precipitates in the transition zone, various approaches have been proposed. Among them there are post-weld heat treatment, adjustment of FSW thermal cycle by increasing the welding speed, application of the backing plate, water as cooling medium, and utilization of additional ultrasonic vibrations [18,19,20,21,22,23]. Although, all of these measures reported positive results in relation to the restored material strength in the transition region, the problem of strengthening precipitates dissolution in the SZ has been still unsolved due to high peak temperature experienced during FSW. Only post-weld heat treatment succeeded to obtain a significant recovery of the joint efficiency [8,18]. However, it is not reliable for the long welds and, moreover, it faces the problem of abnormal grain growth [24,25].

In the present research, the Impulse Friction Stir Welding (IFSW) was utilized as an attempt to increase the mechanical performance of Al-Mg-Si alloy joints by microstructural changes. The principle of the IFSW process is based on the conventional FSW [26]. A distinctive feature of IFSW is an addition of mechanical impulses applied by the vertical movement of the tool, which is the reason that the axial force during the translational movement of the tool is not constant. The sinusoidal variation of the axial force during IFSW leads to the reciprocating motion of the welding tool, enhancing forging action and thereby altering the microstructure in the weld region and the mechanical properties of the IFSW joint. The mechanical impulses are defined by the parameters such as impulse force and frequency. Additional thermal and deformation effect given by impulses is supposed to contribute to the microstructural change, as well as strengthening precipitation behavior in the weld region considering the nature of the base material (BM).

The objective of the current study was to develop a basic understanding of the impact of additional mechanical impulses on the microstructure evolution in the weld with a special focus on strengthening precipitation behavior in the SZ. The effect of microstructural evolution as a result of impulse addition on the overall mechanical properties of IFSW joints has also been elaborated upon.

## 2. Materials and Methods 

The material used in this study was AA 6082-T6 aluminum alloy with the chemical composition given in Table 1. 

The sheets with dimensions of 400 mm × 100 mm × 2 mm were butt welded parallel to the rolling direction using the conventional FSW as well as IFSW. The welding tool used in FSW and IFSW was composed of a smooth concave shoulder with a diameter of 12.5 mm and a smooth cylindrical probe with a diameter and length of 4 and 1.8 mm, respectively. The tilt angle was 2°. All produced joints were performed with a constant rotation speed of 710 rpm (revolution per minute), welding speed of 200 mm/min (millimeter per minute), and impulse force of 2 kN. The impulse frequency was varied from 3 to 10 Hz with an intermediate value of 6 Hz. The FSW welds were produced under position control mode with the heel plunge depth of 0.1 mm. The IFSW joints were welded in the hybrid control mode. The upper position of the welding tool during reciprocating motion was set by the heel plunge depth of 0.1 mm (position control) while the low position of the welding tool was controlled by the impulse force (force control).

The samples for the optical microscopy were automatically cut using a slow speed diamond saw under water cooling and cold mounted in acrylic resins to avoid a thermal influence on the microstructure. After multi-stage polishing, the samples were subjected to a color etching using fresh Weck’s reagent (4 g KMnO_4_, 1 g NaOH, 100 mL distilled water) at 35 °C for ~10–15 s. 

Polished unetched specimens were examined in a scanning electron microscope (SEM) TESCAN MIRA II (Brno, Czech Republic) equipped with an INCA Oxford detector for the energy-dispersive X-ray spectroscopy (EDS) (Tescan, Brno, Czech Republic) to characterize the chemical composition of the constituent particles. Their size was automatically estimated with a detection limit of 0.5 μm. 

The samples for transmission electron microscopy (TEM) were prepared using mechanical thinning down to 100 µm, followed by electrolytic polishing using Struers Tenupol 5 (Cleveland, OH, USA) and reagent containing 10% perchloric acid in butoxyethanol. Finally, ion polishing was carried out using the GATAN DuoMill (Pleasanton, CA, USA) device. The investigations were performed on a Hitachi H-800 (Tokyo, Japan) transmission microscope, equipped with a tungsten filament and an Olympus Quemesa (Münster, Germany) camera. In this research, the operating voltage of 150 kV was used.

Electron backscattered diffraction (EBSD) samples were mounted in epoxy resin, mirror polished, followed by surface preparation using vibratory polisher VibroMet 2 from Buehler (Uzwil, Switzerland) in a LECO solution at RT for 60 min. EBSD was performed using EDAX EBSD system (AMETEK Materials Analysis Division, Mahwah, NJ, USA) with a step size of 0.2 µm. The TSL-OIM (AMETEK Materials Analysis Division, Mahwah, NJ, USA) software was used for the analysis. In each analyzed area, an inverse pole figure (IPF) maps were obtained under the minimum confidence index (CI) of 0.2. In order to eliminate noise effect, a lower-limit boundary misorientation angle of 2° was utilized. Grain size was quantified by applying the grain-reconstruction approach [27]. In this approach, each grain is considered as a circle with equivalent area and calculated the associated circle-equivalent diameter.

The Vickers microhardness was measured along the centerline of the transverse weld section with a load of 0.980 N (HV 0.1) for 10 s after several weeks. The geometry of the tensile specimens (nine specimens of each weld) positioned perpendicular to the welding direction was selected in accordance with DIN 50125 [28]. Tensile tests were carried out up to the rupture of the tested specimens on a tensile machine Walter + bai AG (Löhningen, Switzerland) using 10 mm/min crosshead speed at room temperature. All above-mentioned investigations were carried out several months after welding, taking into account the time-dependent behavior of the studied alloy. Fracture surfaces of the tensile specimens were examined using SEM being gentle cleaned using ultrasonic method for 30 s before observation.

## 3. Results

All welded joints were subjected to the visual examination after welding, in order to reveal the defects such as excessive toe flash, sheet thinning, and surface flaws. Afterwards, the microstructure investigation, microhardness measurements, and tensile tests of the sound FSW and IFSW joints were carried out.

### 3.1. Microhardness and Tensile Behavior

**Figure 1:**
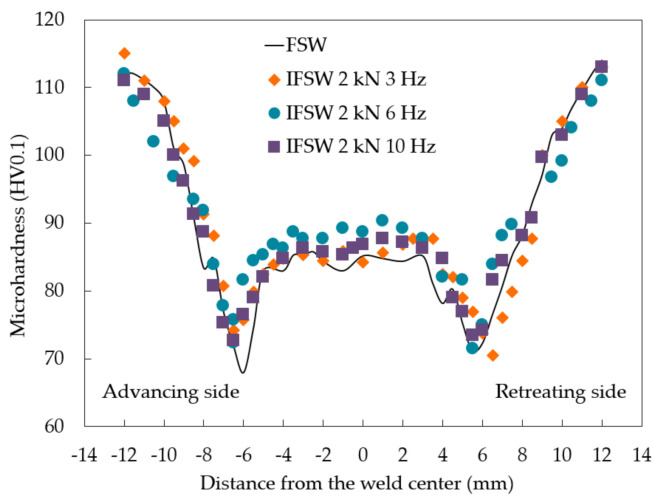
Effect of impulses on the microhardness profiles obtained across the transverse cross-section of the welded joints.

Figure 1 represents the microhardness profiles of three produced welds measured in the centerline of their cross-sections. The microhardness of the BM was 120 HV. As expected, FSW of the precipitation hardenable alloys resulted in an inhomogeneous microhardness distribution implying the formation of the softened region. The minimum hardness value (~70 HV) lied at the distance of 6 mm from the weld center at both advancing (AS) and retreating (RS) sides. No significant difference between the FSW and IFSW microhardness profiles was observed in the SZ, however, the smoothing of the hardness drop in the transition region at the AS with the application of the impulses could be detected.

The overall mechanical properties, such as ultimate tensile stress (UTS), yield stress (YS), and elongation (E), as well as fracture location are summarized in Table 2. It is apparent that UTS and YS were almost the same for the FSW and IFSW joints; however, a positive influence of impulses at lower frequency on elongation can be detected.

It is worth noting that all FSW specimens fractured in the HAZ region on the AS while the fracture location of the IFSW joint varied depending on impulse parameters. Table 2 also provides an amount of the specimens in percentages that broke up in SZ or in the HAZ on AS depending on the parameters. The most interesting aspect is that the value of elongation in the SZ prevailed upon the corresponding values in the HAZ. The influence of impulses on the microhardness and mechanical behavior is discussed in the next sections.

### 3.2. Microstructure Evolution during FSW and IFSW

#### 3.2.1. Base Material (BM)

**Figure 2:**
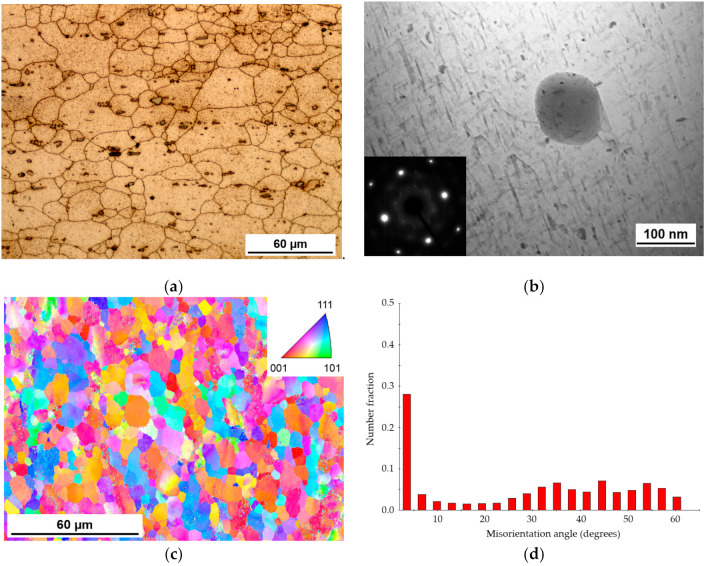
Microstructures of base material: (**a**) Optical image showing the grain microstructure and intermetallic compounds Al_x_Fe_3_MnSi_2_; (**b**) TEM image representing high density of needle-shaped β″ phase with the corresponding diffraction pattern, and Fe-Mn-Si dispersoid; (**c**) EBSD generated IPF map; (**d**) misorientation angle distribution.

The optical microstructure of the BM is presented in Figure 2. The grain structure in the transverse direction of the wrought base sheet was dominated by slightly elongated grains (Figure 2a). The average grain size was 38 µm along and 20 µm across the rolling direction. Besides, the structure of the BM contained the coarse intermetallic particles with an average diameter of up to 12 µm that are evenly distributed inside and along grain boundaries and aligned along the rolling direction. Using the EDS measurement, it was found that these particles acquired an average chemical composition Al_x_Fe_3_MnSi_2_ with a varied Fe content. 

The TEM image (Figure 2b) of the BM demonstrated a high density of needle-like precipitates, which are suggested to be a metastable β″ precipitates according to their morphology, size, and the corresponding selected area electron diffraction (SAED) pattern with streaks along the <100> direction of aluminum. The presence of streaks may result from the different orientation of the needle-type precipitates (presumably β″). The presence of the coherent fine needles of β″ phase contributing predominantly to the strengthening of such alloys is widely reported in the literature, e.g., [6,8]. Along with β″ precipitates, coarse precipitates of round morphology have been found to be randomly dispersed in the microstructure. These particles with a diameter over 100 nm were identified to be rich in Al, Mn, and Si that allowed presuming that they are dispersoids. This type of precipitate is not considered to directly contribute to the alloy strengthening, but they can be a nucleation site for the hardening precipitates [13].

According to EBSD results, grain structure of initial AA 6082-T6 aluminum alloy is inhomogeneous with the size ranging from a few to dozens of microns. This is a microstructure similar to that of a typical rolled plate of aluminium alloy. Misorientation distribution is plotted in Figure 2d. The structure of the BM dominated by HAGBs that is typical of wrought alloys. In inverse pole figure (IPF) orientation map, individual grains are colored according to their crystallographic orientations relative to the welding direction with an orientation code triangle. IPF map shows that there is no preferred orientation of grains in BM.

#### 3.2.2. Microstructure in the Stir Zone 

As was mentioned in the introduction, the current research focused on the investigation of the microstructural changes that occurred in the SZ of the IFSW joints. For the closed observation, the joints produced with 3 and 10 Hz were chosen as extreme points. 

**Figure 3:**
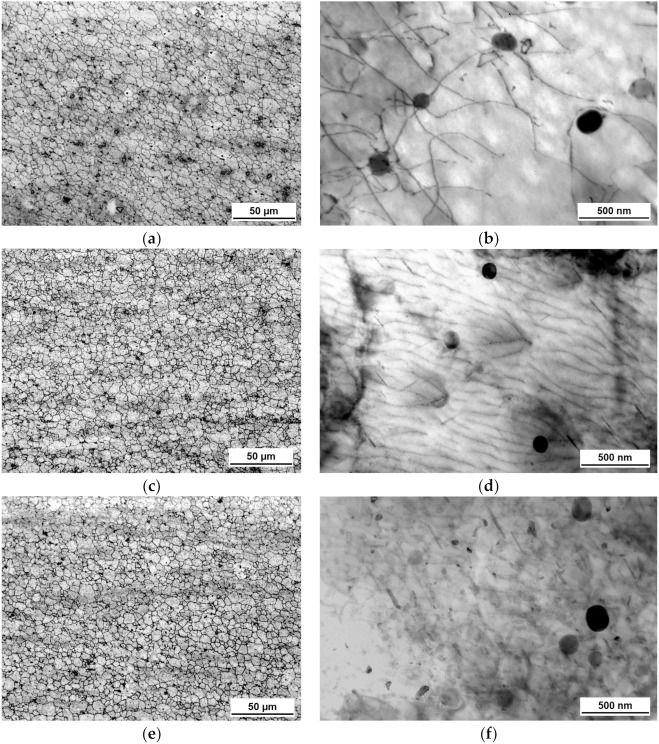
Microstructure in Stir Zone FSW weld (**a**) optical image; (**b**) TEM image; IFSW 2 kN 3 Hz weld (**c**) optical image; (**d**) TEM image; IFSW 2 kN 10 Hz weld; (**e**) optical image; (**f**) TEM image.

The grain microstructures of the produced joints obtained via optical microscopy are presented in Figure 3a,c,e. After FSW as well as IFSW, fine equiaxed grains were achieved as a result of dynamic crystallization, and they were significantly smaller than those in the BM. It can be seen that the SZ of the impulse welds possessed more homogeneous grains distribution, compared to the FSW structure. Coarse intermetallic particles of Al-Fe-Mn-Si inherited from the BM structure were also visible in the microstructures of the SZ, indicating their insoluble nature or only partial dissolution, considering slight decrease in their average size. 

Figure 3b shows the precipitate morphology and distribution in the SZ of the FSW weld. It demonstrated round particles with an average diameter in the range of 100–120 nm which were also observed in the microstructure of the BM (Figure 2b). This observation indicated that Fe-Mn-Si dispersoids were stable to survive the thermal load during the FSW cycle as they remained in the structure with preserved size and morphology. Moreover, the formation of Orowan loops when the dislocations interacts with these particles, as well as their shearing by dislocations evidenced coherent and semi-coherent interaction of the dispersoids with a matrix. That also means that they contributed to the strengthening of the alloy through the precipitation hardening mechanism. No presence of the β″ precipitates was detected. 

Conversely, Figure 3d,f demonstrated the fine needles in size range of 80–120 nm aligned in different orientations, and also the presence of few randomly distributed coarse round particles. The morphology of needle-shaped precipitates indicated that they are β″ type, though the comparison between their distribution in the SZ and the BM structure (Figure 2b) revealed less density of the β″ precipitates in the weld. Nevertheless, partial reprecipitation of the hardening particles during natural aging occurred by impulse utilization. Coarse round particles are supposed to be Fe-Mn-Si dispersoids. However, they were free of dislocations in case of IFSW in comparison with FSW. Dislocation walls aligned in a subgrains boundary are visible in Figure 3f. Therefore, different kinds of dislocation structures were observed in the SZ of the FSW and IFSW welds. It supports an assumption that completion of the recovery and dynamic recrystallization processes had reached different grades without and with impulses introduction into the weld structure. 

#### 3.2.3. EBSD Analysis in the Stir Zone 

**Figure 4:**
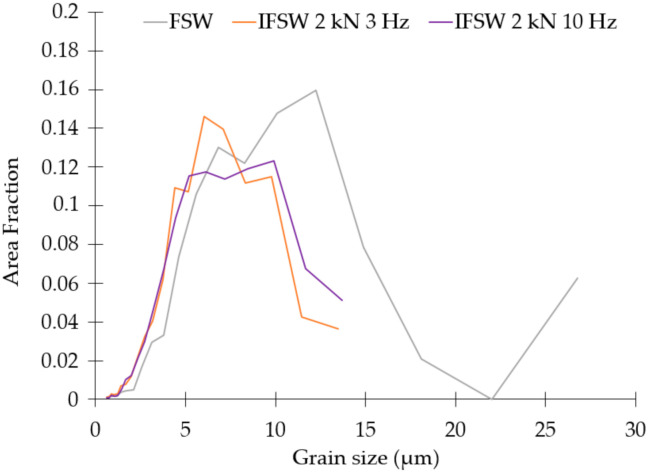
Grain size distributions of the produced FSW and IFSW welds.

A significant grain refinement in the SZ achieved by impulses in comparison with the conventional FSW process was evident on optical images (Figure 3a,c,e). This conclusion was confirmed by the grain size distributions obtained by Electron Backscatter Diffraction (EBSD) analysis, as demonstrated in Figure 4. IFSW produced a fine-grained equiaxed microstructure in the SZ; the grain size decreased up to 5 µm for IFSW joints in comparison to the conventional FSW with 12 µm grain size. It is worth noting that the samples processed at 3 and 10 Hz had almost the same average grain size, but narrower grain size distributions compared with the FSW profile displaying the homogeneity of the grain structure in the SZ. Such evidence can be explained by the further results of EBSD analysis.

**Figure 5:**
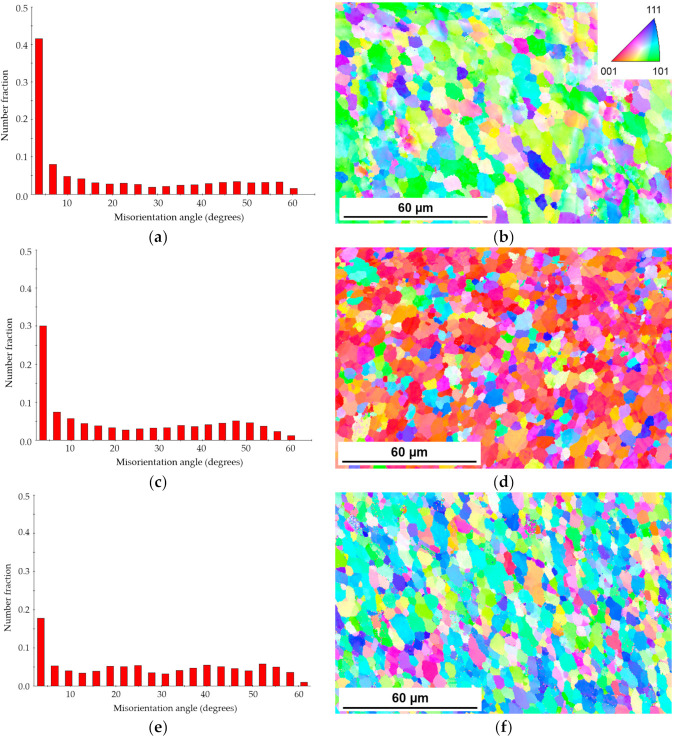
Misorientation angle distribution and EBSD maps in the SZ of AA6082-T6 joints: (**a**,**b**) FSW; (**c**,**d**) IFSW 2 kN 3 Hz; (**e**,**f**) IFSW 2 kN 10 Hz.

The fraction of the low-angle grain boundaries (LAGBs) and high-angle grain boundaries (HAGBs) as well as the histograms of the misorientation angle distribution for the SZ of the studied joints are summarized in Table 3, and Figure 5a,c,e, respectively. In this work, 15° misorientation was utilized to distinguish between the HAGBs and LAGBs. Closer examination of Table 3 shows that the fraction of LAGBs decreased from 0.591 for FSW to 0.314 while the fraction of HAGBs increased to 0.686 at the higher frequency. The fraction of HAGBs in the IFSW 3 Hz weld with a misorientation angle between 40 and 60° augmented significantly (Figure 5). In contrast, microstructural changes in the IFSW 10 Hz weld tend to be dominated by the formation of HAGBs in the wide range of misorientation angle between 20 and 60°. These observations indicated that the transformation of the LAGBs into HAGBs occurred more effectively under impulse influence. 

Figure 5b,d,f depict the IPF maps with the color code taken from the SZ center of the studied FSW and IFSW joints. IPF map of the FSW joint showed an accumulation of grains with <101> orientation (welding direction). Application of 3 Hz impulse led to a significant change in preferred orientation of <001> with a very low amount of grains with other orientations. Further pulse frequency increase results in orientation in <111> and <122> directions. 

#### 3.2.4. Fractography 

**Figure 6:**
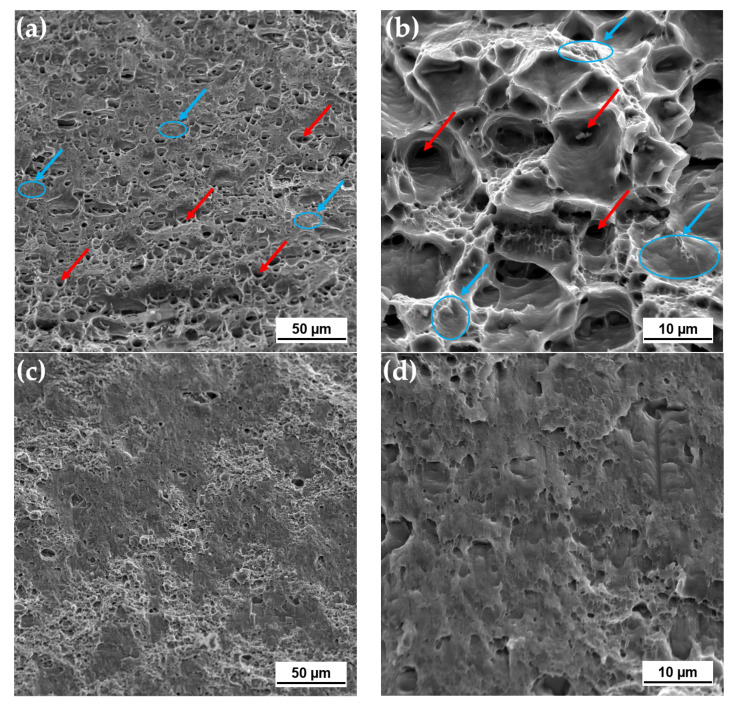

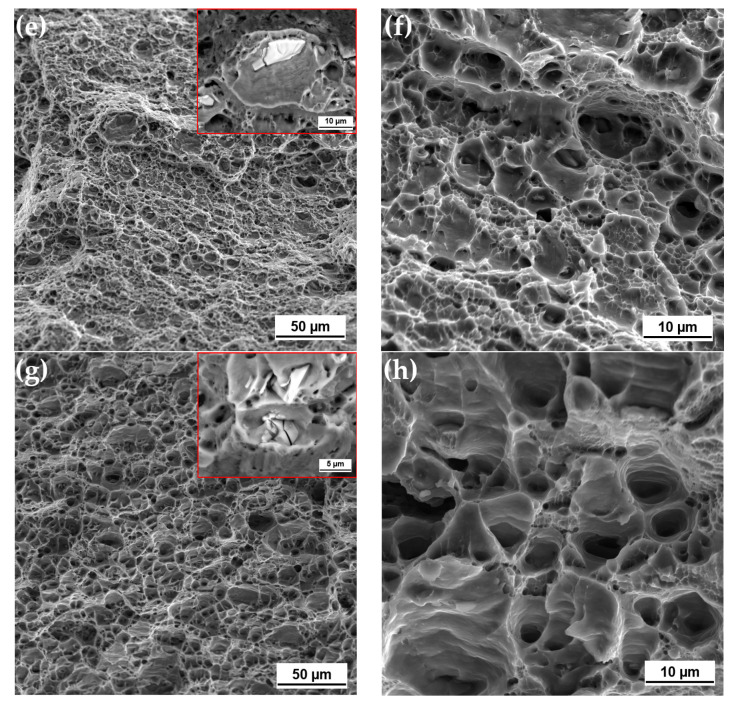
Fracture topography of AA6082-T6 joints: (**a**) FSW—HAZ (×1000). Dominant ductile fracture with cleavage/quasicleavage fracture areas; (**b**) FSW (×5000). Equiaxial dimples, visible large microvoids with surrounding many fine dimples; (**c**) IFSW 2 kN 3 Hz—center of the SZ (×1000). Ductile-cleavage fracture; (**d**) IFSW 2 kN 3 Hz (×5000). Cleavage fracture, visible cleavage steps; (**e**) IFSW 2 kN 3 Hz—surrounding region of the SZ center (×1000). Mostly ductile fracture with broken intermetallic inclusions (BSE); (**f**) Magnification of region (**e**) (×5000); (**g**) IFSW 2 kN 10 Hz—HAZ (×1000). Mostly ductile fracture with broken intermetallic inclusions (BSE); (**h**) IFSW 2 kN 10 Hz (×5000). Large microvoids with many surrounding fine dimples.

Scanning electron fractographs of the studied joints at two different magnifications (×1000 and ×5000) are presented in Figure 6. As mentioned above, the fracture of all tested FSW tensile samples located in the HAZ region close to the original joint line on the AS. Fracture surface was inclined at approximately 45% to the tensile load axis. As can be seen form Figure 6a,b, the FSW fracture surface represented the features of both ductile and brittle failures. The ductile mode expressed in unevenly distributed dimples up to about 10 μm in diameter (some of them are indicated with red arrows in Figure 6a,b), while the brittle topography had cleavage and quasicleavage fracture areas (some of them are represented as blue areas marked with blue arrows in Figure 6a,b). Nevertheless, the ductile failure mode was predominant.

In IFSW joints at 3Hz, fracture occurred in 40% cases in the center of the SZ and in 60% near to the joint line. The fractographic features of the welds that broke up in the HAZ are not presented since they possessed similar features to the FSW surface. It should be noted that the specimens fractured in the SZ were defect-free, and their fracture surfaces were also 45° shear fractures to the tensile direction. Conversely, the fracture surfaces of the welds, which fractured in the SZ, could be divided into two zones. In the center of the weld there was is a region of about 1.2 mm width with features of the cleavage and quasi-cleavage fracture such as cleavage steps (Figure 6c,d). This brittle zone was surrounded by ductile fracture surfaces that mostly consisted of a large number of shallow, deep, and uniform dimples, as presented in Figure 6e,f. Therefore, despite the fact that the fracture surface of the 3 Hz samples was a combination of dimple and brittle rupture, the contribution of the dimple mode prevailed due to the features of the constituents. Notably, the 6 Hz specimens that also fractured partially in the SZ demonstrated similar fracture behavior. 

Further increase in impulse frequency led to a fracture only in the HAZ similar to the welds without impulse. The fracture surface of the 10 Hz joints was characterized by dominated ductile fracture; cleavage cracking features were identified locally (Figure 6g,h). However, the size of the dimples significantly increased in comparison to the ductile surface of the 3 Hz joint. The homogeneity of the dimple distribution improved significantly with impulse utilization. 

An interesting observation was made related to the morphology of the intermetallic particles presented in the IFSW fracture surfaces, which are the Al-Fe-Mn-Si second phase particles inherited from the BM (identified as Al_x_Fe_3_MnSi_2_ in the BM). The morphological features of these particles can influence the rupture behavior since they act as dimple nucleation sites. In the surfaces of the IFSW welds, the broken particles were observed (Figure 6e,g) using BSE (backscattered electron) method. This kind of observation supports an assumption that additional plastic strain introduced by impulses in FSW may cause the breakage of the brittle particles. The fragmentation of some intermetallics can contribute to the ductility increase, as the small particles result in the formation of equiaxed fine dimples homogenously distributed in the matrix. 

## 4. Discussion

### 4.1. Microstructural Evolution during IFSW

It is reported that higher peak temperature leads to grain coarsening in the SZ of the FSW welds while larger plastic deformation causes smaller grain size [4,17]. Whereas, there is an evidence that strain rate is a dominating factor regarding the grain refinement [29,30]. According to the numerical model of the temperature distribution in FSW and IFSW [31], the maximum temperature achieved in the SZ of the IFSW joint (2 kN, 3 Hz) was almost the same as for the conventional FSW process. It was also demonstrated that a further increase in the impulse frequency had no significant influence on the peak temperature in the SZ. The heating and the cooling rates during FSW and IFSW were also identical because the welding speed remained constant. Therefore, the influence of plastic deformation (strain and strain rate) during IFSW seemed to be a prevailed factor in terms of grain size in the SZ so that temperature impact was excluded from the explanation. This was also a reason for ignoring a possible influence of post-dynamic recrystallization on the grain structure evolution. 

It is generally considered that FSW promotes the dynamic recrystallization process in the SZ that implies in the earlier stage the formation of subgrains via pile-up of dislocations introduced by plastic deformation. The increase in subgrains boundary misorientation by the continuous accumulation and absorption of dislocations results in the gradual evolution of the subgrains to HAGBs by rotation. When the misorientation angle between the rotated subgrains exceeds 15°, a new grain, which size is bigger than the preceding subgrains, is formed. It is known that the kinetics of these processes is accelerated by increasing deformation temperature and require very large strain [1,4,8,32,33]. 

The results of the EBSD analysis have shown that the SZ of the FSW weld contained a higher fraction of LAGBs compared to the IFSW joints. Moreover, the TEM image of the FSW SZ (Figure 3b) represented dislocation tangles within the subgrains when interacting with second phase particles. It pointed out that the recrystallization process during FSW has not fully completed since there are free moving dislocations in the structure of the FSW joint. In contrast, the structure of the impulse SZ contained dislocations walls arranged at the subgrains boundaries. It could be considered as a step forward towards to a complete formation of the grain. These findings, combined, suggest that microstructure evolutions are promoted with impulse utilization. Furthermore, a significantly higher fraction of HAGBs in the SZ of IFSW welds supported this assumption, taken into account that the grain refinement happened with the accumulation of LAGBs. 

As mentioned above, the recrystallization evolution in the SZ by FSW depends on the temperature as well as induced strain and strain rate. It is assumed that the use of impulses entails increased strain compared to conventional FSW resulting in two factors favorable for the recrystallization process. Firstly, increased strain accelerates accumulated dislocation density that supports in turn development of subgrains network. Secondly, additional strain generated by mechanical impulses could advance the rotation of subgrains. This may be attributed to facilitation of dislocation glide by additional plastic deformation. Rotation and grow of subgrains gave rise to the formation of equiaxed recrystallized grains with high-angle boundaries. This mechanism was proposed in the research [8,30]. Contribution of these factors due to impulses enhanced the homogeneity of the recrystallized microstructure with a small grain size.

### 4.2. Precipitation Evolution during IFSW

As reported in the introduction, reprecipitation of the strengthening β″ precipitates in the SZ after FSW hardly occurs during subsequent natural aging due to high cooling rate or incomplete dissolution of atoms responsible for hardening precipitation formation. The results of the current investigation coincided with the majority of the previous findings reporting the absence of the β″ particles in the SZ of the FSW weld as a result of their dissolution not followed by reversion (Figure 3b). In contrast, the β″ precipitates were found in the SZ microstructure of the IFSW joints, even if their density was significantly lower compared to the BM structure. It is known that the precipitation phenomena strongly depend on the thermo-mechanical cycle experienced during FSW, including temperature and plastic deformation. Peak temperature, as well as cooling rate were not affected by impulses that excluded the first factor from the explanation of the β″ reversion. In addition, since a full dissolution of β″ was evident in case of FSW (Figure 3b), the same behavior can be attributed to the IFSW process, considering almost the same thermal cycle. Moreover, it is a supporting aspect in relation to the complete dissolution of the atoms responsible for age hardening. 

The second contributor, plastic deformation, is inextricably linked to dislocation density. In the case of FSW, dislocations play a key role as the mechanism of precipitates formation in the SZ is heterogeneous meaning their nucleation at the subgrain boundaries and network of dislocations [8]. The dislocations promote the diffusion of the solute atoms that is a driven force for the precipitation formation. This effect has been reported by Frigaard et al. [34]. More developed subgrain network as well as high dislocation density introduced by impulses could accelerate precipitation kinetics followed by their nucleation and growth during natural aging. The reprecipitation of hardening precipitates may also contribute to the grain refinement in the SZ of the IFSW welds since nucleation and coarsening of coherent particles occurs on subgrains boundaries and dislocations. Under these conditions, precipitates can inhibit grain growth due to the pining effect. 

### 4.3. Mechanical Behavior of the IFSW Joints

No significant difference between the FSW and IFSW microhardness profiles was observed in the SZ (Figure 1). It is well-established that four microstructural characteristics of precipitation hardenable aluminum alloys, such as grain size, solute atoms, dislocation density, and precipitates, contribute to the material strength. Grain boundary strengthening is described by the Hall-Petch relationship [35] as follows,

$$\sigma_{GB}=\sigma_{0}+kd^{-1/2}$$

where *σ*_0_ is the friction stress, *k* is the Hall-Petch slope, *d* is the effective grain size. Considering that *k* is equal to 0.04 MPa·m^1/2^ [35] and *d* alternates up to two times only (Figure 4), the impact of the grain refinement on the microhardness of the SZ is extremely low and could be neglected. Since solid solution strengthening was the same for the FSW and IFSW, the microhardness level in SZ cannot be connected to this strengthening mechanism.

The dislocation density, as demonstrated in the Section 3.2.2, was high in the SZ of the FSW joint. Moreover, the formation of Orowan loops when the dislocations interact with the second phase particles as well as their shearing by dislocations was observed. In contrast, no free dislocations were found in the SZ of the IFSW joints, as they were consumed for the formation of the subgrain boundaries. This led to the stress relaxation and, consequently, to a decrease in strength. Obviously, the contribution of the dislocation structures to strengthening was higher in case of FSW compared to IFSW. The precipitation hardening took part in strengthening of the IFSW welds since the partial reprecipitation of the fine needle-shaped β″ type precipitates occurred during natural aging by impulse utilization.

To summarize, the impact of different strengthening mechanism on the microhardness level in the SZ of the FSW and IFSW joints, it could be suggested that similar profiles formed as a result of two competitive mechanisms. The microhardness of the FSW weld was increased by dislocation tangles while the reprecipitated the β″ reversion was the main contributor in case of IFSW. 

As can be seen from the microhardness profiles (Figure 1), the minimum hardness levels were detected at the distance of 6 mm from the weld center at both AS and retreating RS after FSW and IFSW. However, the hardness decrease in the transition region at the AS was smoothed by impulses. This fact may explain the shift of the fracture location from the HAZ, typical for the FSW joints, to the SZ for the IFSW joints at 3 and 6 Hz, considering the defect-free SZ of the IFSW joints. However, it cannot be the main reason for the fracture in the SZ since the difference in the microhardness was not significant. This shift could be also a consequence of the change in grain orientation as a result of additional material flow caused by impulses. Such observations were previously reported by Sato et al. [36] who suggested that fracture location of the 6xxx-FSW joints could be related to the distribution of the crystallographic grain orientation as well as strain tensor of the applied deformation. Similar, the abnormal fracture in the weld nugget was detected in the 7xxx series high-strength aluminum alloy joints produced by FSW [37]. The lowest average Taylor factor, the largest area fraction of grains with high strain, and inhomogeneous strain concentration in the nugget were claimed to be responsible for such fracture behavior. Presumably, the phenomenon of the shift in the fracture location is related to material flow and intermixing during the IFSW process. The utilization of impulses seems to result in the additional material flow along the welding tool axis while the material in the SZ during conventional FSW flows predominantly in tangential direction around the tool probe. This additional material flow leads to the change of the grain orientation. Figure 5 shows that the application of 3 Hz impulse resulted in the preferred orientation of <001>; further increase in impulse frequency to 10 Hz changed the orientation to <111> and <122> directions compared to <101> orientation for the FSW weld. The orientation directions in case of IFSW at 10 Hz are preferable for a slip direction in fcc-structures. The relationship between the fracture behavior, material flow, and the distribution of the crystallographic grain orientation during IFSW will be studied in the further investigation. 

As can be seen from Table 2, the IFSW specimens raptured partially in the SZ possessed the higher elongation. Firstly, it could be related to the tortuous grain boundaries due to smaller grain size hindering the joints failure [38]. Secondly, the results of the fractographic investigation have shown that the surface of the 3 Hz and 6 Hz joints contained two regions; one of them consisted of smaller and shallower dimples than those of FSW and IFSW at 10 Hz. This demonstrated ductile the behavior of the material, which was probably caused by the breakage of the intermetallic particles by impulses.

## 5. Conclusions

AA6082-T6 aluminum alloy of 2.0 mm in thickness was subjected to impulse friction stir welding (IFSW) by various impulse frequencies, as well as friction stir welding (FSW). The aim of these experiments was to gain some understanding on effect of mechanical impulses on the microstructural and precipitation evolution in the stir zone (SZ), and, consequently, on the mechanical properties of the IFSW joints.

(1) It was shown that the application of impulses in the FSW process promoted the dynamic recrystallization in the SZ in two ways: Accumulation of higher number of dislocations compared to the conventional FSW, and acceleration of the subgrains rotation. It led to the formation of the fine homogeneous grain microstructure.
(2) The results of TEM study indicated that impulses entailed the partial re-reprecipitation of the strengthening β″ precipitates in the SZ of the IFSW joints.
(3) The alignment of the microhardness profiles after FSW and IFSW was attributed to the contribution of different strengthening mechanisms such as dislocation and precipitation hardening. On the one hand, the re-precipitation of β″ phase in IFSW process increased the microhardness in the SZ of the IFSW joints; on the other hand, the developed dislocation network in the FSW joint strengthened the matrix.
(4) Increased elongation of the IFSW joints at comparable levels of the tensile and yield stress in comparison with the joints produced by FSW was caused by the breakage of the intermetallic particles, the grain refinement, and the change of the grain orientation. The shift in the fracture location of the IFSW joints from the HAZ, which is considered to be typical for the FSW joints of the precipitation hardenable aluminum alloys, to the SZ could be related to material flow and intermixing change through impulses.

It should be noted that the present research focused on the investigation in the SZ since the precipitation phenomena in the Al-Mg-Si after FSW, studied in the previous research, had some contradictory points. The second reason was an assumption that impulses act in the SZ so that they can alter the microstructure and precipitation behavior in this region. The further experiments will be conducted in order to investigate other regions of the IFSW joints, especially the softened region where the effect of impulses were detected. 

## Figures and Tables

**Table 1:** Chemical composition of Aluminum Alloy 6082 (wt%) used in the study.

Si	Mg	Cu	Mn	Fe	Al
0.89	1.18	0.31	0.4	0.4	Bal.

**Table 2:** Mechanical properties of the studied joints, tensile test.

Specimen	UTS (MPa)	YS (MPa)	E (%)	Fracture Location
FSW	237.2 ± 4.8	162.9 ± 2.9	4.0 ± 0.1	HAZ (100%)
IFSW 2 kN 3 Hz	236.7 ± 2.6	165.2 ± 5.1	5.3 ± 0.4	SZ (40%); HAZ (60%)
IFSW 2 kN 6 Hz	239.4 ± 9.8	161.6 ± 9.6	5.2 ± 0.5	SZ (80%); HAZ (20%)
IFSW 2 kN 10 Hz	239.2 ± 3.2	163.8 ± 2.2	3.9 ± 0.11	HAZ (100%)

**Table 3:** Fraction of the LAGBs and HAGBs defined in the Stir Zone of the studied joints.

Specimen	Fraction LAGBs (2–15°)	Fraction HAGBs (15–180°)
FSW	0.591	0.409
IFSW 2 kN 3 Hz	0.480	0.520
IFSW 2 kN 10 Hz	0.314	0.686

## Data Availability

Data available on request due to restrictions eg privacy. The data presented in this study are available on request from the corresponding author.
